# Organosolv pretreatment of sorghum bagasse using a low concentration of hydrophobic solvents such as 1-butanol or 1-pentanol

**DOI:** 10.1186/s13068-016-0427-z

**Published:** 2016-02-02

**Authors:** Hiroshi Teramura, Kengo Sasaki, Tomoko Oshima, Fumio Matsuda, Mami Okamoto, Tomokazu Shirai, Hideo Kawaguchi, Chiaki Ogino, Ko Hirano, Takashi Sazuka, Hidemi Kitano, Jun Kikuchi, Akihiko Kondo

**Affiliations:** Department of Chemical Science and Engineering, Graduate School of Engineering, Kobe University, 1-1 Rokkodaicho, Nada-ku, Hyogo Kobe, 657-8501 Japan; Organization of Advanced Science and Technology, Kobe University, 1-1 Rokkodaicho, Nada-ku, Hyogo Kobe, 657-8501 Japan; Department of Bioinformatic Engineering, Graduate School of Information Science and Technology, Osaka University, 1-5 Yamadaoka, Osaka Suita, 565-0871 Japan; RIKEN Center for Sustainable Resource Science, 1-7-22 Suehiro-cho, Tsurumi-ku, Kanagawa Yokohama, 230-0045 Japan; Bioscience and Biotechnology Center, Nagoya University, 1 Furo-cho, Chikusa-ku, Nagoya, 464-8601 Japan; Graduate School of Medical Life Science, Yokohama City University, 1-7-29 Suehirocho, Tsurumi-ku, Yokohama, 230-0045 Japan; Graduate School of Bioagricultural Sciences and School of Agricultural Sciences, Nagoya University, 1 Furo-cho, Chikusa-ku, Nagoya, 464-8601 Japan

**Keywords:** Sorghum bagasse, Organosolv pretreatment, Fractionation, 1-butanol, 1-pentanol, Lignin

## Abstract

**Background:**

The primary components of lignocellulosic biomass such as sorghum bagasse are cellulose, hemicellulose, and lignin. Each component can be utilized as a sustainable resource for producing biofuels and bio-based products. However, due to their complicated structures, fractionation of lignocellulosic biomass components is required. Organosolv pretreatment is an attractive method for this purpose. However, as organosolv pretreatment uses high concentrations of organic solvents (>50 %), decreasing the concentration necessary for fractionation would help reduce processing costs. In this study, we sought to identify organic solvents capable of efficiently fractionating sorghum bagasse components at low concentrations.

**Results:**

Five alcohols (ethanol, 1-propanol, 2-propanol, 1-butanol, and 1-pentanol) were used for organosolv pretreatment of sorghum bagasse at a concentration of 12.5 %. Sulfuric acid (1 %) was used as a catalyst. With 1-butanol and 1-pentanol, three fractions (black liquor, liquid fraction containing xylose, and cellulose-enriched solid fraction) were obtained after pretreatment. Two-dimensional nuclear magnetic resonance analysis revealed that the lignin aromatic components of raw sorghum bagasse were concentrated in the black liquor fraction, although the major lignin side-chain (β-O-4 linkage) was lost. Pretreatment with 1-butanol or 1-pentanol effectively removed *p*-coumarate, some guaiacyl, and syringyl. Compared with using no solvent, pretreatment with 1-butanol or 1-pentanol resulted in two-fold greater ethanol production from the solid fraction by *Saccharomyces cerevisiae*.

**Conclusions:**

Our results revealed that a low concentration (12.5 %) of a highly hydrophobic solvent such as 1-butanol or 1-pentanol can be used to separate the black liquor from the solid and liquid fractions. The efficient delignification and visible separation of the lignin-rich fraction possible with this method simplify the fractionation of sorghum bagasse.

**Electronic supplementary material:**

The online version of this article (doi:10.1186/s13068-016-0427-z) contains supplementary material, which is available to authorized users.

## Background

The depletion of fossil fuels and environmental pollution associated with fossil fuel use have increased interest in the utilization of biomass as a feedstock for the production of biofuels and bio-based chemicals [1, 2]. Starch-rich biomass is one of the main feedstocks used for these purposes; however, this results in direct competition with global food supplies. Lignocellulosic biomass is a promising alternative feedstock because it is abundant, inexpensive, and renewable. Sorghum (*Sorghum bicolor* L. Moench), which is a highly productive C4 photosynthetic plant, can be used to produce lignocellulosic fractions. Sorghum is also attractive because it uses water efficiently and is drought tolerant [3]. The lignocellulosic fraction produced from sorghum is typically referred to as sorghum bagasse.

The primary components of sorghum bagasse are cellulose, hemicellulose, and lignin [4], each of which can be valorized. For example, cellulose and hemicellulose are used as carbohydrate sources for fermentation, whereas cellulose pulp is used for paper, and lignin is a renewable source of aromatics [5–7]. The fractionation of sorghum bagasse into its primary components can be carried out in a biorefinery [5, 8]. However, because cellulose is highly crystalline in nature, exhibits complex chemical cross-linking between components, and is recalcitrant to hydrolysis due to the sheathing by hemicellulose and lignin, the fractionation of sorghum bagasse requires appropriate pretreatment [9, 10]. Organosolv pretreatment is a practical methodology for fractionating sorghum bagasse because it facilitates the isolation of high-quality lignin and high-purity cellulose [2, 11, 12]. Organosolv pretreatment involves the use of high-concentration (30–70 %) organic or aqueous organic solvents at temperatures of 100–200 °C, with or without the addition of catalysts [13]. Thus, organosolv pretreatment is expensive and necessitates the recovery of the organic solvents used. Decreasing the concentration of organic solvent necessary for effective pretreatment would thus reduce the cost of fractionation.

As indicated above, organosolv pretreatment has the advantage of enabling the isolation of lignin [2]. The composition of plant cell walls can be characterized by two-dimensional ^1^H-^13^C heteronuclear single-quantum coherence nuclear magnetic resonance (2D ^1^H-^13^C HSQC NMR) spectroscopy [14, 15]. Thus, 2D-NMR spectroscopy can be used to elucidate the fate of lignin during organosolv pretreatment of sorghum bagasse.

The aim of the present study was to investigate the fractionation effectiveness of five different organic solvents (ethanol, 1-propanol, 2-propanol, 1-butanol, and 1-pentanol) at a relatively low concentration (12.5 %) in organosolv pretreatment of sorghum bagasse. Sulfuric acid was used as the catalyst. The detailed structures of the lignin products obtained in the black liquor and solid fraction of samples pretreated with 1-butanol or 1-pentanol were characterized using 2D ^1^H-^13^C HSQC NMR spectroscopy.

## Results and discussion

### The influence of solvent type on organosolv fractionation of sorghum bagasse

Sorghum bagasse was fractionated into three primary components important in biorefinery processes: cellulose, hemicellulose, and lignin. In this study, fractionation was evaluated by comparing pretreatment using five different organic solvents (ethanol, 1-propanol, 2-propanol, 1-butanol, and 1-pentanol) at a low concentration (12.5 %). Pretreatment with no addition of solvent was used as a control.

**Fig. 1:**
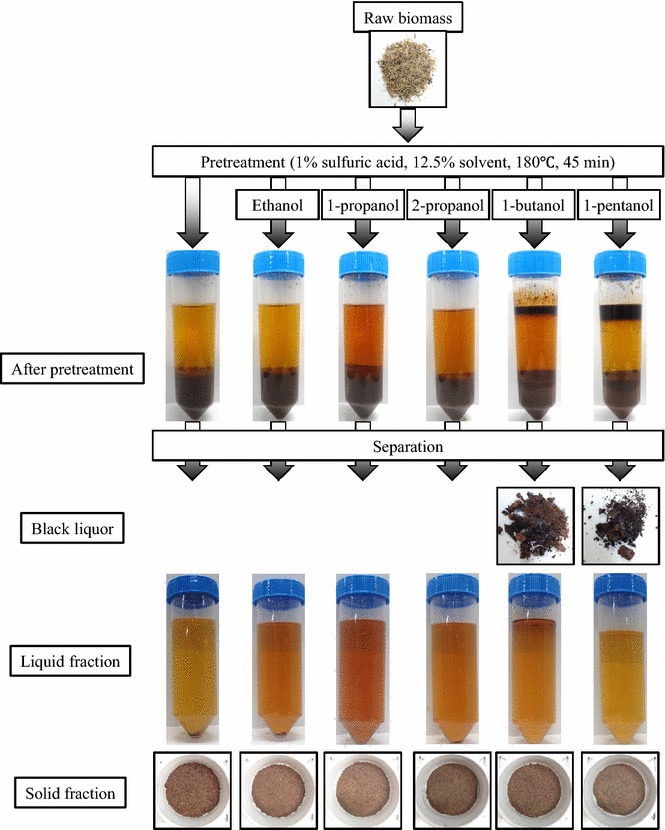
Organosolv pretreatment of sorghum bagasse using 12.5 % solvent concentration. Ethanol, 1-propanol, 2-propanol, 1-butanol, and 1-pentanol were used as the solvent. No addition of solvent was the control. Using ethanol, 1-propanol, and 2-propanol as the solvent and no solvent (control), a solid fraction and a liquid fraction were obtained. Using 1-butanol and 1-pentanol as the solvent, a black liquor fraction was obtained in addition to solid and liquid fractions

After treatment at 180 °C for 45 min, samples were centrifuged (Fig. 1). Interestingly, three fractions (solid, liquid, and black liquor) were obtained with 1-butanol or 1-pentanol as the solvent, but only two fractions (solid and liquid) were obtained using ethanol, 1-propanol, or 2-propanol as the solvent and when no solvent was used (control). These results suggest that differences in the physiochemical properties of the solvents affect fractionation. The greater hydrophobicity of 1-butanol and 1-pentanol (partition coefficients: log*P*_ow_ = 0.88 and 1.51, respectively) compared with ethanol, 1-propanol, and 2-propanol (log*P*_ow_ = −0.31, 0.25, and 0.05, respectively) resulted in clearer fractionation when using the former solvents. The control condition was equal to dilute acid pretreatment [16]. Thus, hydrophobic 1-butanol and 1-pentanol separated the black liquor fraction from the acid solution even at the low concentration of 12.5 %.

### Cellulose-enriched solid fraction

**Fig. 2:**
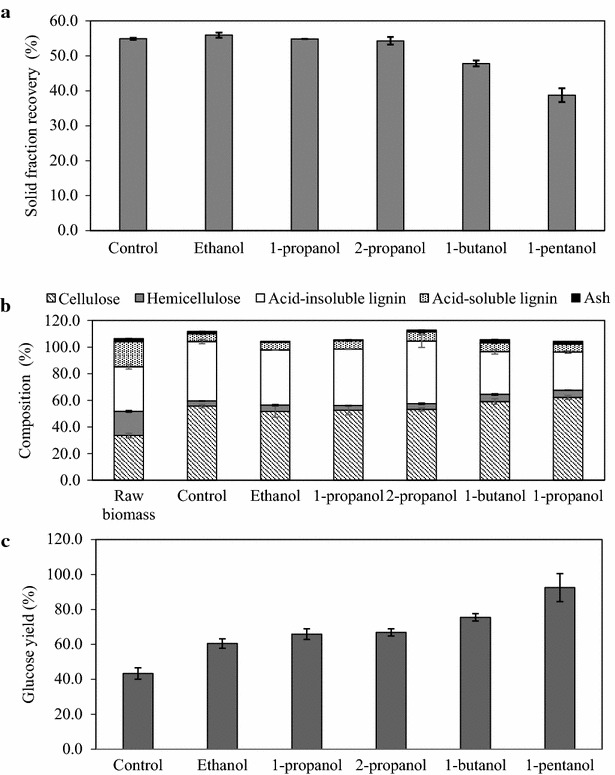
Properties of the solid fraction obtained after organosolv pretreatment. **a** Weight of the solid fraction obtained after pretreatment. **b** Carbohydrate and lignin composition of the solid fraction. **c** Glucose yield from the solid fraction. Organosolv pretreatment of sorghum bagasse was carried out using ethanol, 1-propanol, 2-propanol, 1-butanol, and 1-pentanol as the solvent and no solvent (control)

To clarify the effect of solvent type on raw sorghum bagasse, the solid fraction obtained after pretreatment was characterized. Compared with the control, the dry weight of the solid fraction decreased when 1-butanol or 1-pentanol was used as the solvent, but ethanol, 1-propanol, and 2-propanol had no effect on solid fraction dry weight (Fig. 2a). Cellulose is reportedly enriched in the solid fraction of samples pretreated using acid [9], and an increase in the cellulose content in all solid fractions was observed compared with the raw biomass (Fig. 2b). In particular, the cellulose content was higher in the solid fraction when 1-butanol or 1-pentanol was used as the solvent (59.1 and 62.2 %, respectively) compared with ethanol, 1-propanol, 2-propanol, or no solvent (control) (51.6–55.6 %). Cellulose recovery in the solid fraction ranged from 84.4 to 91.1 % when ethanol, 1-propanol, 2-propanol, or 1-butanol was used as the solvent and when no solvent was used (control) (Additional file  1). Cellulose recovery in the solid fraction was 71.9 % when 1-pentanol was used as the solvent. Similar to the trend with dry weight (Fig. 2a), the acid-insoluble lignin content was lower in the solid fraction when 1-butanol or 1-pentanol was used as the solvent compared with other solvents (Fig. 2b). Accordingly, the greatest increase in glucose yield compared with the control was observed with 1-butanol or 1-pentanol as the solvent, but increased glucose yield was also observed with ethanol, 1-propanol, and 2-propanol (Fig. 2c).

Lignin removal, as calculated using the equation [(lignin in bagasse)—(lignin in solid fraction)]/(lignin in bagasse), was higher when 1-butanol or 1-pentanol was used as the solvent (64.7–74.3 %), compared with the use of no solvent, ethanol, 1-propanol, or 2-propanol (44.1–49.7 %) (Fig. 2a, b). Lignin, which is degraded by sulfuric acid, is reportedly liberated from the cell wall and forms droplets that attach to cellulose molecules [17]. Hydrophobic alcohols would dissolve these lignin droplets and prevent their attachment to cellulose. The removal of lignin from cellulose reportedly increases the accessibility to hydrolytic enzymes and reduces the degree of irreversible adsorption of the enzyme to lignin [18]. Thus, organosolv pretreatment using 1-butanol or 1-pentanol efficiently removes acid-insoluble lignin, resulting in enhanced enzymatic hydrolysis of cellulose.

### Liquid fraction containing hemicellulose-derived sugars

**Fig. 3:**
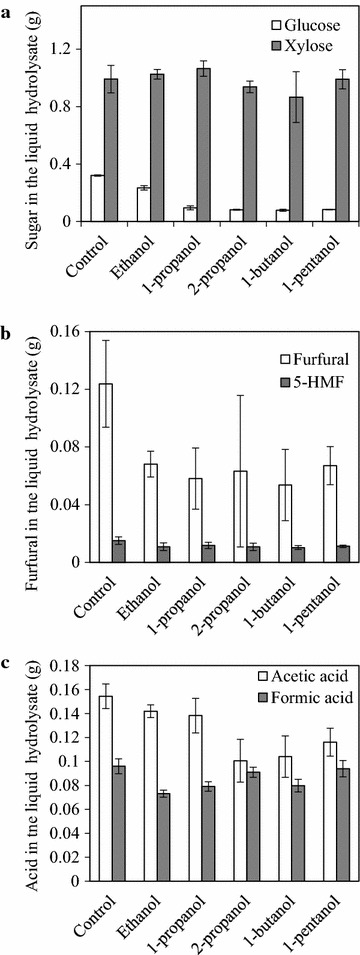
Composition of the liquid fraction. **a** Glucose and xylose. **b** Furfural and 5-HMF. **c** Acetic acid and formic acid. The amount (g) of each component obtained from 6 g of raw biomass is shown

It was expected that hemicellulose-derived sugars, mainly xylose, would be contained in the liquid fraction [2]. The amount of xylose contained in the liquid fraction of samples pretreated with ethanol, 1-propanol, 2-propanol, 1-butanol, 1-pentanol, or with no solvent (control) was similar (Fig. 3a). The xylose recovery in the liquid fraction was higher (90.6–97.4 %) when using ethanol, 1-propanol, 1-pentanol, or no solvent, compared with 2-propanol and 1-butanol (85.5 and 79.2 %, respectively), as calculated using the following equation: (xylose amount in liquid fraction)/[(total xylose)—(xylose amount in solid fraction)]. The reason for the decreased xylose recovery when using 2-propanol or 1-butanol as the solvent is unclear.

Compared with the control, the amount of glucose in the liquid fraction was lower in the solvent-pretreated samples (Fig. 3a). Furfural and 5-hydroxymethylfurfural (5-HMF) were contained in the liquid fraction (Fig. 3b). The harsh conditions of pretreatment resulted in the production of similar amounts of acetic and formic acids in the liquid fraction (Fig. 3c). In addition, solvents were blended in the liquid fraction (data not shown). The presence of acetic and formic acids, furfural, 5-HMF, and the pretreatment solvent would inhibit subsequent fermentation. These fermentation inhibitors could be removed, and the xylose and glucose in the liquid fraction could be concentrated, by subsequent application of membrane separation nanofiltration [19, 20].

### Lignin contained in the black liquor fraction

**Fig. 4:**
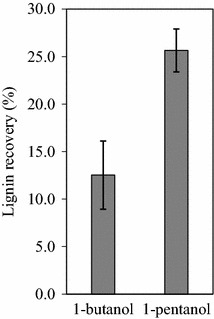
Yield of lignin in black liquor. Lignin recovery was calculated as follows: (lignin in black liquor)/[(lignin in raw biomass)—(lignin in solid fraction)]

A black liquor fraction was obtained when 12.5 % 1-butanol or 1-pentanol was used as the solvent (Fig. 1). Lignin was precipitated from the black liquor fraction by dilution with water [2]. Lignin recovery, as calculated using the equation (lignin in black liquor)/[(lignin in raw biomass)—(lignin in solid fraction)], was 12.5 and 25.6 % using 1-butanol or 1-pentanol as the solvent, respectively, corresponding to 8.1 and 19.1 % lignin content in the raw sorghum bagasse, respectively (Fig. 4). The lignin recovery in the present study was similar to that of a previous study, which reported 5.1–17.2 % lignin recovery from wheat straw pretreated with 50–60 % ethanol at 190–210 °C [5]. However, our lignin recovery was lower than that from rye straw pretreated at 190 °C for 3 h at a 1.3 % acid concentration (35 %) [21]. Therefore, process optimization of lignin recovery with respect to solvent concentration, temperature, time, and acid concentration will be necessary in the future. It is also possible that low lignin recovery was due to the loss of water-soluble products from lignin degradation [21].

**Fig. 5:**
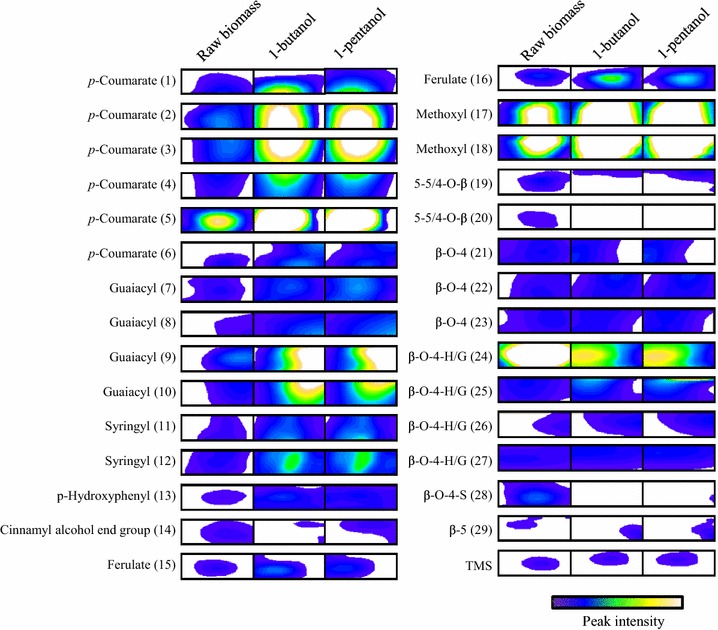
Peaks of raw sorghum bagasse and black liquor. *Left* portion of figure shows NMR peaks of raw biomass. *Middle* and *right* portions of figure show NMR peaks of black liquor obtained after organosolv pretreatment of sorghum bagasse using 1-butanol or 1-pentanol as the solvent, respectively

Next, the composition and structure of the lignins in the black liquor fraction were analyzed using 2D ^1^H-^13^C HSQC NMR [15]. The syringyl, guaiacyl, and *p*-hydroxyphenyl lignin units are important aromatic elements of the plant cell wall [22]. Strong signals related to these lignin components (regions of interest [ROIs] 7–13) were detected in the black liquor, compared with raw sorghum bagasse (Fig. 5 and Additional file 2). Other strong signals related to aromatic components, such as *p*-coumarate (ROIs 1–6) and ferulate (ROIs 15 and 16), were detected in the black liquor. The only aromatic region signal that was weaker in the black liquor was that related to cinnamyl alcohol (ROI 14). Accordingly, signals related to methoxyl (ROIs 17 and 18) groups were stronger, as methoxyl groups are found in the side chains of syringyl, guaiacyl, and ferulate [23].

In contrast, the signal related to a major interunit structure, β–O–4 (ROI 24), was weaker in the black liquor than in raw sorghum bagasse. This was probably due to partial cleavage of the thermally labile β–O–4 unit in raw sorghum bagasse under heat treatment [24, 25]. Another signal (ROI 25) related to the β–O–4 unit was stronger in the black liquor, but the reason for this difference is unclear. Signals related to other minor interunit structures in raw sorghum bagasse were either stronger or weaker in the black liquor. These results suggest that most of the lignin aromatic components were concentrated in the black liquor in samples pretreated using 1-butanol or 1-pentanol as the solvent; however, the major linkage structure was lost.

**Fig. 6:**
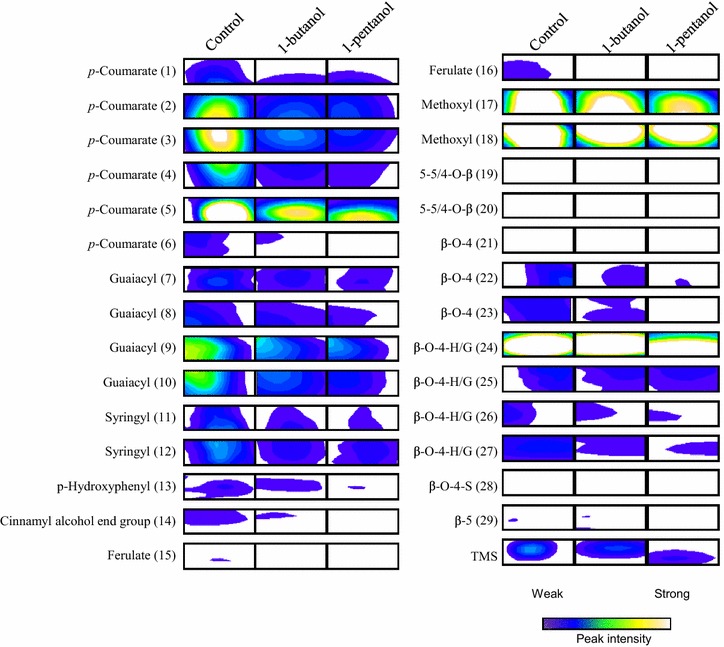
Peak of solid fractions. *Left*, *middle*, and *right* portions of figure show NMR peaks of the solid fractions obtained by organosolv pretreatment of sorghum bagasse using no solvent (control) and 1-butanol and 1-pentanol as the solvent, respectively

Which lignin constituent is most affected by 1-butanol or 1-pentanol was also investigated by comparing 2D NMR signals of solid fractions obtained by pretreatment with no solvent (control), 1-butanol, or 1-pentanol (Fig. 6 and Additional file 3). Compared with the solid fraction obtained using no solvent (control), signals related to *p*-coumarate (ROIs 2–6) were weaker in solid fractions obtained using 1-butanol or 1-pentanol as the solvent. In addition, the signals related to guaiacyl (ROI 10), syringyl (ROIs 11 and 12), and minor β–O–4 interunits (ROIs 21–23, 26, and 27) in solid fractions obtained using 1-butanol or 1-pentanol as the solvent were weaker compared with the control. Accordingly, because the guaiacyl, syringyl, and β–O–4 units contain a methoxyl side chain, methoxyl-related signals (ROIs 17 and 18) were weaker in solid fractions obtained using 1-butanol or 1-pentanol [23]. The removal of *p*-coumarate, syringyl, and guaiacyl from the solid fraction using 1-butanol or 1-pentanol corresponded with the results of increased aromatic lignin regions in the black liquor fraction and decreased acid-insoluble content in the solid fraction.

### Ethanol fermentation using the solid fraction

**Fig. 7:**
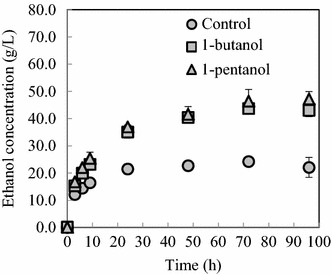
Simultaneous saccharification and fermentation of solid fractions by *Saccharomyces cerevisiae*. Solid fractions were obtained by organosolv pretreatment of sorghum bagasse using 1-butanol (*closed square*) and 1-pentanol (*closed triangle*) as the solvent and no solvent (*closed circle*). Ethanol fermentation was initiated from 200 g/L of solid fraction

The solid fraction obtained after organosolv pretreatment can be utilized as a sugar source for microbial fermentation. The solid fractions obtained after organosolv pretreatment using 1-butanol or 1-pentanol as the solvent were subjected to simultaneous saccharification and fermentation by *S. cerevisiae*. Ethanol production using each of these solid fractions was compared with that obtained with control solid fraction (Fig. 7). As expected, the ethanol production rates obtained with solid fractions of samples pretreated with 1-butanol or 1-pentanol (2.6 and 2.8 g/L/h, respectively) were about 1.6 times higher in the first 9 h than those obtained with the solid fraction of samples pretreated with no solvent (1.6 g/L/h). After 96 h of fermentation, ethanol production from the 1-butanol and 1-pentanol solid fractions was about twice that of the control, reaching 43.1 and 47.2 g/L, respectively. In addition, the theoretical ethanol yield from the 1-butanol and 1-pentanol solid fractions was 59.6 and 62.1 %, respectively, compared with 32.4 % for the control. As suggested previously [26], organosolv delignification and the resultant supply of readily hydrolyzable cellulose substrates results in an increase in the concentration of microbial fermentation products.

## Conclusions

Sorghum bagasse was fractionated by organosolv pretreatment using various solvents at a low concentration of 12.5 %. The catalyst for the reaction was 1 % sulfuric acid, and the reaction was carried out at 180 °C for 45 min. Pretreatment with the highly hydrophobic solvents 1-butanol and 1-pentanol produced three visible fractions (i.e., a cellulose-enriched solid fraction, a liquid fraction containing hemicellulose-derived xylose, and a black liquor fraction containing lignin). However, only two fractions were produced upon pretreatment with ethanol, 1-propanol, or 2-propanol (i.e., a solid fraction containing cellulose and a liquid fraction containing xylose), and recovery of lignin was difficult. 2D NMR spectroscopy revealed that the black liquor fraction contained lignin aromatic components, such as guaiacyl, syringyl, *p*-hydroxyphenyl, *p*-coumarate, and ferulate. The aliphatic β–O–4 unit of lignin was absent in the black liquor fraction. Pretreatment with 1-butanol or 1-pentanol effectively removed *p*-coumarate, syringyl, and some of the guaiacyl from raw sorghum bagasse. The 1-butanol or 1-pentanol should be recovered after pretreatment and recycled using separation technologies such as pervaporation [27, 28]. We demonstrated here the effective organosolv pretreatment of sorghum bagasse using a low concentration of hydrophobic solvent (1-butanol or 1-pentanol).

## Methods

### Plant materials

The hybrid sorghum cultivar Tentaka [29] was grown in 2013 at an experimental field in Okinawa, Japan. Whole plants were harvested at the heading stage and then fully dried in a greenhouse. After removal of the panicles, the culms were ground into a fine powder using a blender (WB-1; TGK, Hachioji, Japan) fitted with a 2-mm screen.

### Organosolv pretreatment

Organosolv pretreatment was performed using a laboratory-scale thermostirrer (HHE-19G-U; Koike Precision Instruments, Kanagawa, Japan) having a total volume of 100 mL. Sorghum bagasse powder (6 g) was suspended in 80 mL of aqueous solution. The aqueous solution included 70 mL of 1 % sulfuric acid and 10 mL of solvent (ethanol, 1-propanol, 2-propanol, 1-butanol, or 1-pentanol). An aqueous solution consisting of 80 mL of 1 % sulfuric acid was used as the control. Next, the mixture was treated at 180 °C for 45 min, with agitation at 200 rpm. The optimal temperature and agitation speed for pretreatment were determined according to a previous report [30]. After pretreatment, the mixture was centrifuged at 3500 rpm for 10 min for fractionation. Solid and liquid fractions were obtained under the control condition and with ethanol, 1-propanol, or 2-propanol (Fig. 1). When using 1-butanol or 1-pentanol as the solvent, the supernatant including the liquid and black liquor fractions was recovered and again centrifuged at 3500 rpm for 10 min in order to separate the fractions. The upper phase contained the black liquor fraction, and the lower phase contained the liquid fraction. The solid fraction was washed with deionized water, neutralized to pH 7.0, then dried; the dry weight was measured using an electronic balance (XS105DU; Mettler Toledo, Greifensee, Switzerland). Lignin was separated from the black liquor fraction by precipitation upon dilution with 80 mL of water. The compositions of cellulose, hemicellulose, acid-insoluble lignin, acid-soluble lignin, and ash were determined according to the National Renewable Energy Laboratory method [31].

### Sugar analysis

Sugar analysis was performed as described previously [30]. The liquid fraction was neutralized to pH 5.0 by the addition of calcium hydroxide. An aliquot of sample (1.5 μL) was mixed with 1.5 μL of 0.1 % (w/w) Ribitol as internal standard, and then the mixture was dried in a vacuum concentrator (7810010; Labconco, Kansas City, MO, USA). The dried residue was dissolved in 100 μL of 20 mg/mL of methoxyamine hydrochloride in pyridine and incubated at 30 °C for 90 min, after which 50 μL of *N*-methyl-*N*-trimethylsilyltrifluoroacetamide was added and the sample was incubated at 37 °C for 30 min. A 10 μL aliquot of the solution was subjected to gas chromatography-mass spectrometry (GC–MS) (GCMS-2010plus; Shimadzu, Kyoto, Japan) under the following conditions: column, Agilent CP-Sil 8CB-MS (30 m × 0.25 mm); carrier gas, helium; injection temperature, 230 °C; oven temperature, 80 °C at *t* = 0–2 min, then increased to 330 °C at 15 °C/min.

### Byproduct analysis

Byproduct analysis was performed as described previously [30]. Acetone (900 μL) was added to 100 µL of liquid fraction and mixed thoroughly. The sample was then centrifuged at 21,880×*g* and room temperature for 10 min. The supernatant (10 µL) was subjected to GC–MS analysis (GC-MS-2010plus; Shimadzu). 5-HMF and furfural were analyzed under the following conditions: column, Agilent CP-Sil 8CB-MS (30 m × 0.25 mm); carrier gas, helium; injection temperature, 250 °C; oven temperature, 50 °C at *t* = 0–5 min, then increased to 280 °C at 20 °C/min. Acetic acid and formic acid were analyzed under the following conditions: column, Agilent DB-FFAP (60 m × 0.25 mm); carrier gas, helium; injection temperature, 250 °C; oven temperature, 100 °C at *t* = 0 to 5 min, then increased to 230 °C at 10 °C/min.

### Enzymatic saccharification

Enzymatic saccharification of the solid fraction (10 % dry weight) was performed by adding 0.3 M citrate buffer (pH 4.8) and cellulase (Celic CTec2, Novozyme, Bagsvaerd, Denmark) at a concentration of 6.6 filter paper units (FPU)/g-dry biomass. Tetracycline (40 μg/mL) and cycloheximide (30 μg/mL) were added to prevent microbial growth. The reaction mixture was incubated at 50 °C in a chemi station (PPS-2000, Tokyo Rikakikai, Tokyo, Japan) with agitation at 120 rpm for 72 h. Enzymatic saccharification was stopped by rapid chilling on ice, followed by centrifugation at 21,880×*g* for 10 min at 4 °C. The sugars in the supernatant were analyzed as described above.

### Composition analysis

Compositional analysis was performed according to the National Renewable Energy Laboratory (NREL) method [31]. Obtained sugar was analyzed as described above. The amount of acid-soluble lignin was determined by measuring the optical density (OD) at 240 nm.

### Solution 2D NMR spectroscopy

Sample preparation for solubilized lignocelluloses was similar to previously reported methods [32, 33]. Briefly, the dried sample was further ground using a Pulverisette 5 ball mill (Fritsch GmbH, Idar-Oberstein, Germany). The ball-milled powder (30 mg) was mixed with 600 µL of dimethyl sulfoxide (DMSO)-d_6_:pyridine-d_5_ (4:1), heated at 50 °C for 30 min in a Thermomixer Comfort (Eppendorf AG, Hamburg, Germany), then centrifuged at 20,380×*g* for 5 min. The supernatant was transferred to 5-mm ϕ NMR tubes and subjected to NMR analysis. NMR spectra were recorded on an Avance III HD-600 (Bruker, Billerica, MA, USA) equipped with a 5-mm TXI-cryoprobe operated at 600 MHz for ^1^H- and 125 MHz for ^13^C-NMR. The temperature of all NMR samples was maintained at 319 K. The chemical shifts were referenced to the methyl group of DMSO-d_6_ at ^13^C = 40.03 ppm and ^1^H = 2.582 ppm.

Two-dimensional ^1^H-^13^C heteronuclear single-quantum spectra were collected using echo/antiecho gradient selection (the hsqcetgp pulse program in the Bruker library). Forty-eight regions of interest (ROIs) were compared with previously assigned chemical shifts [23, 34]. ROI details are described in Additional file 4.

### Liquefaction and ethanol fermentation

Liquefaction of the solid fraction was performed in a 50 mL polypropylene tube (Corning Inc., NY, USA) set in a Thermo Block Rotator SN-06BN heat block (Nissin, Tokyo, Japan), as described previously [35]. The tube was closed with a silicone plug (As One, Osaka, Japan), into which a hole was made using a disposable needle (φ = 0.6 mm) (Termo Corp., Tokyo, Japan). In the tube, the solid fraction (final weight 200 g/L) was mixed with medium containing 50 mM citric acid buffer (pH 5.0) and 6.6 FPU/g-biomass of commercial cellulase (Cellic CTec2; Novozymes Inc., Bagsvaerd, Denmark) and axially rotated at 35 rpm at 50 °C for 2 h.

*Saccharomyces cerevisiae* strain BY4741 (genotype: *MAT*a *his3*∆*1leu2*∆*0met15*∆*0ura3*∆*0*) [36] was purchased from the American Type Culture Collection (ATCC) as ATCC No. 404002. The cells were aerobically propagated for 24 h at 30 °C and 150 rpm in 5 mL of YPD medium [10 g/L yeast extract [Becton, Dickinson and Company, Tokyo, Japan], 20/g L polypeptone (Wako Pure Chemical Industries, Ltd., Osaka, Japan), and 20 g/L glucose], and then cultivated for 24 h in 500 mL of YPD medium. The cells were collected by centrifugation at 3000×*g* for 10 min at 4 °C and washed twice with distilled water. Ethanol fermentation was initiated by the addition of yeast extract (final concentration 10 g/L), peptone (final concentration 20 g/L), and *S. cerevisiae* cells (50 g-wet cells/L, corresponding to 10 g-dry cells/L) after 2 h of liquefaction of the solid fraction. Ethanol fermentation was conducted by axial agitation at 35 rpm and 35 °C for 48 h. Theoretical ethanol yield (%) was calculated as the percent of 0.511 g-ethanol/g-sugar of consumed glucose as follows:

$${\text{Ethanol}}\,{\text{yield}}\,{ = }\,\frac{{{\text{Ethanol}}\,{\text{produced}}\,\left( {\text{g}} \right)}}{{{\text{Glucan}}\,{\text{in}}\,{\text{solid}}\,{\text{fraction}}\left( {\text{g}} \right)}}\,$$

## Additional files

10.1186/s13068-016-0427-z Mass balance of glucose, xylose and lignin. Black bar, lined bar, gray bar, open bar and dotted bar indicated recovery in solid fraction, liquid fraction, liquid fraction byproduct, black liquor and unknown. (A) Mass balance of glucose. Liquid fraction byproduct and black liquor mean 5-HMF. (B) Mass balance of xylose. Liquid fraction byproduct and black liquor mean furfural. (C) Mass balance of lignin.
10.1186/s13068-016-0427-z Peak intensities obtained by 2D NMR analysis of raw sorghum bagasse and black liquor. Black liquor was obtained after organosolv pretreatment of sorghum bagasse using 1-butanol or 1-pentanol as the solvent. Blue bar indicates peak intensity of raw sorghum bagasse. Orange and green bars indicate peak intensities of the solid fraction obtained using 1-butanol and 1-pentanol as the solvent, respectively.
10.1186/s13068-016-0427-z Peak intensities obtained by 2D NMR analysis of solid fractions. The solid fractions were obtained by organosolv pretreatment of sorghum bagasse using 1-butanol and 1-pentanol as the solvent and no solvent (control). Red, orange, and blue bars indicate solid fraction pretreated with no solvent, 1-butanol, and 1-pentanol, respectively.
10.1186/s13068-016-0427-z Annotation of each 2D ^1^H-^13^C HSQC NMR spectrum peak.

## Abbreviations

2D ^1^H-^13^C HSQC NMR: two-dimensional ^1^H-^13^C heteronuclear single-quantum coherence nuclear magnetic resonance
ROI: region of interest
5-HMF: 5-hydroxymethylfurfural
GC–MS: gas chromatography-mass spectrometry
FPU: filter paper units
