# Supplementary material for: Organosolv pretreatment of sorghum bagasse using a low concentration of hydrophobic solvents such as 1-butanol or 1-pentanol
Source: Biotechnol Biofuels. 2016 Feb 2;9:27. doi: 10.1186/s13068-016-0427-z (PMC4736640; doi:10.1186/s13068-016-0427-z)
Supplement: Supplementary file 1 — 10.1186/s13068-016-0427-z Mass balance of glucose, xylose and lignin. Black bar, lined bar, gray bar, open bar and dotted bar indicated recovery in solid fraction, liquid fraction, liquid fraction byproduct, black liquor and unknown. (A) Mass balance of glucose. Liquid fraction byproduct and black liquor mean 5-HMF. (B) Mass balance of xylose. Liquid fraction byproduct and black liquor mean furfural. (C) Mass balance of lignin. [file 13068_2016_427_MOESM1_ESM.pptx]

## Slide 1
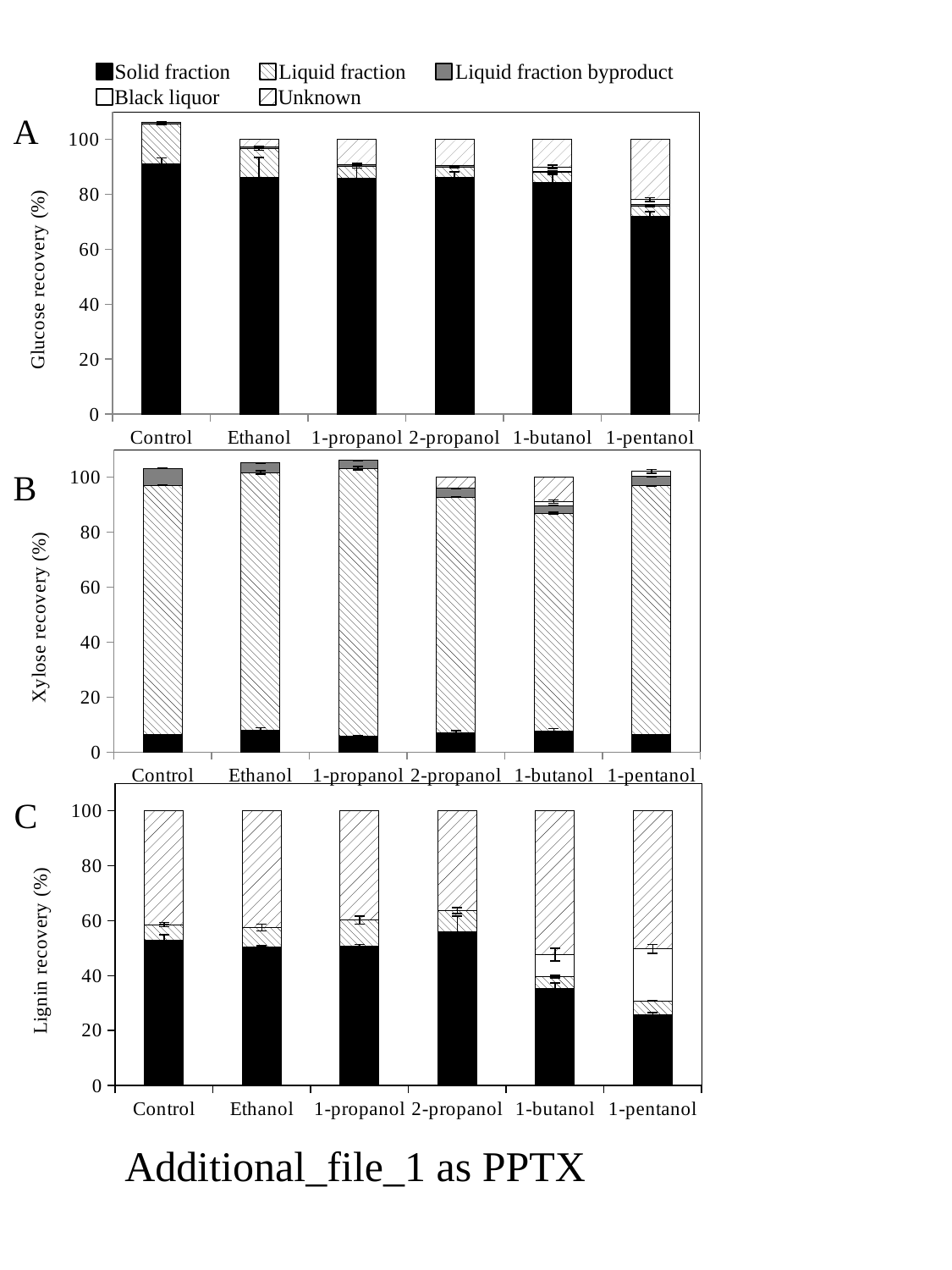

Solid fraction
Liquid fraction
Liquid fraction byproduct
Black liquor
Unknown
A
### Chart
| Category | | | | Black liquor | |
|---|---|---|---|---|---|
| Control | 91.10418719572692 | 14.343801746242063 | 0.7557993849622331 | 0.0 | 0.0 |
| Ethanol | 86.18712249089965 | 10.505219301728182 | 0.545601293365243 | 0.0 | 2.762056914006921 |
| 1-propanol | 85.8867696509655 | 4.290122821355648 | 0.5862615985436064 | 0.0 | 9.23684592913524 |
| 2-propanol | 86.13660362607767 | 3.683195137960374 | 0.5408653394183347 | 0.0 | 9.639335896543619 |
| 1-butanol | 84.37537661821521 | 3.5252139817077124 | 0.5139443175863724 | 1.5380838156222276 | 10.047381266868475 |
| 1-pentanol | 71.91556527837676 | 3.739412922106198 | 0.5598977453049565 | 1.889046780918687 | 21.8960772732934 |
### Chart
| Category | | | | | |
|---|---|---|---|---|---|
| Control | 6.368829542048933 | 90.75392458874799 | 6.155942503097288 | 0.0 | 0.0 |
| Ethanol | 7.931747056245167 | 93.8272612339744 | 3.3894856501778445 | 0.0 | 0.0 |
| 1-propanol | 5.846924797870017 | 97.41180288733143 | 2.8960835439751293 | 0.0 | 0.0 |
| 2-propanol | 6.943717294800137 | 85.83131659526937 | 3.146849767588359 | 0.0 | 4.078116342342133 |
| 1-butanol | 7.6720388339185845 | 79.26364489739794 | 2.6718591881948686 | 1.5380838156222276 | 8.854373264866387 |
| 1-pentanol | 6.250661051757729 | 90.63565847652136 | 3.33718901562998 | 1.889046780918687 | 0.0 |B
### Chart
| Category | Solid fraction | | Black liquor | |
|---|---|---|---|---|
| Control | 52.8368338233071 | 5.725915875169551 | 0.0 | 41.43725030152335 |
| Ethanol | 50.34222071460829 | 7.174355495250947 | 0.0 | 42.48342379014076 |
| 1-propanol | 50.81718537991774 | 9.37924016282216 | 0.0 | 39.8035744572601 |
| 2-propanol | 55.88960924543981 | 7.805291723202094 | 0.0 | 36.3050990313581 |
| 1-butanol | 35.323460820894724 | 4.249355495250977 | 8.10401119402979 | 52.323172489824515 |
| 1-pentanol | 25.674431582617217 | 4.96415366350063 | 19.062913415874995 | 50.298501338007156 |C
Additional_file_1 as PPTX
