# Supplementary material for: Organosolv pretreatment of sorghum bagasse using a low concentration of hydrophobic solvents such as 1-butanol or 1-pentanol
Source: Biotechnol Biofuels. 2016 Feb 2;9:27. doi: 10.1186/s13068-016-0427-z (PMC4736640; doi:10.1186/s13068-016-0427-z)
Supplement: Supplementary file 2 — 10.1186/s13068-016-0427-z Peak intensities obtained by 2D NMR analysis of raw sorghum bagasse and black liquor. Black liquor was obtained after organosolv pretreatment of sorghum bagasse using 1-butanol or 1-pentanol as the solvent. Blue bar indicates peak intensity of raw sorghum bagasse. Orange and green bars indicate peak intensities of the solid fraction obtained using 1-butanol and 1-pentanol as the solvent, respectively. [file 13068_2016_427_MOESM2_ESM.pptx]

## Slide 1
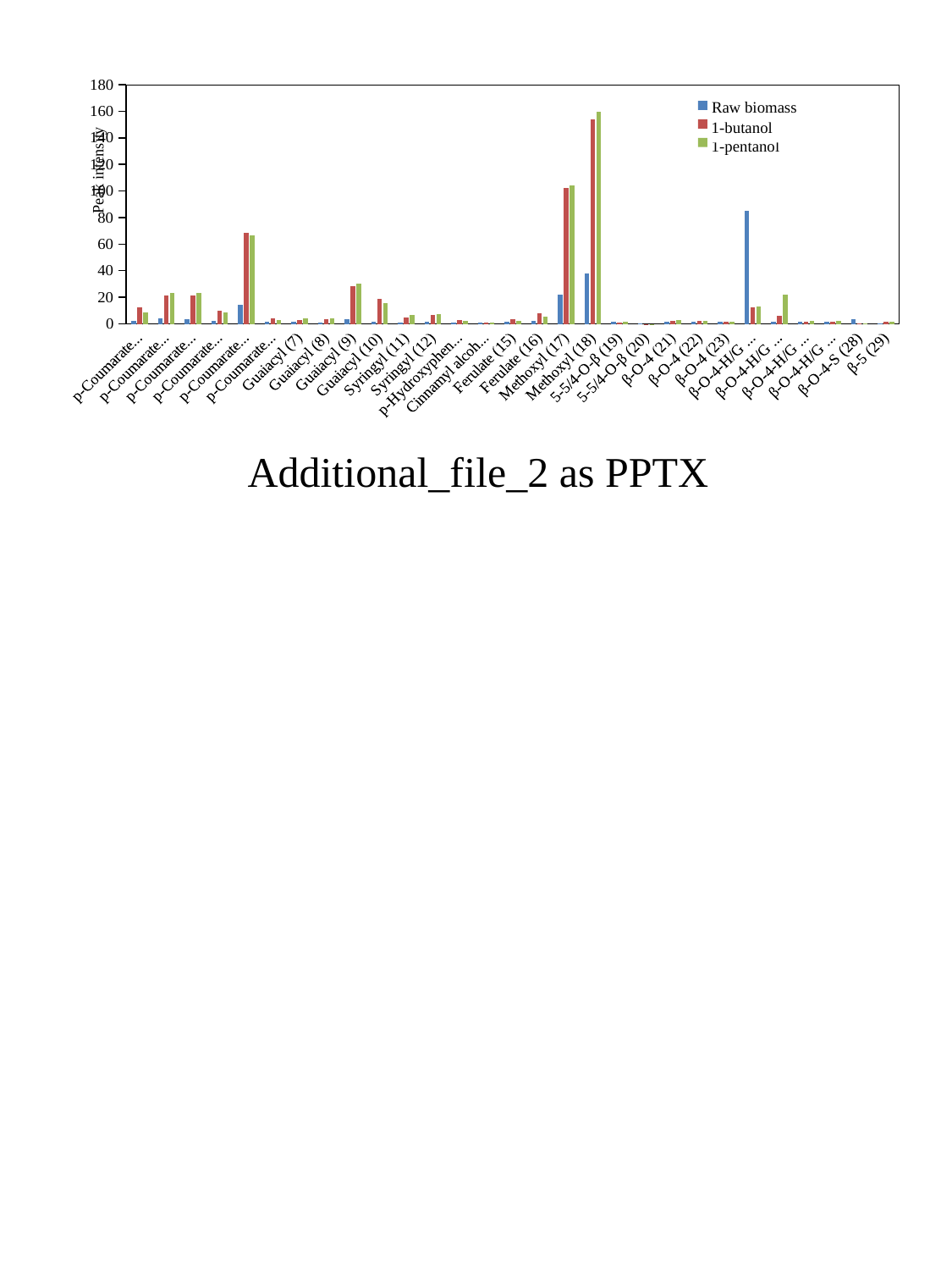

### Chart
| Category | | | |
|---|---|---|---|
| p-Coumarate (1) | 2.248505143830263 | 12.714256688968819 | 8.860259323177267 |
| p-Coumarate (2) | 3.884811880055664 | 21.438594124114847 | 23.519428545414666 |
| p-Coumarate (3) | 3.7650784283199785 | 21.438594124114847 | 23.519428545414666 |
| p-Coumarate (4) | 2.273089252921403 | 10.177212353332678 | 8.923492627126711 |
| p-Coumarate (5) | 14.281018662667675 | 68.59701593816784 | 66.51903538068692 |
| p-Coumarate (6) | 1.4695297954541122 | 4.193129611679021 | 2.8779194550323015 |
| Guaiacyl (7) | 1.4255530650111519 | 2.9812268195506193 | 4.27314022737337 |
| Guaiacyl (8) | 0.832538777299791 | 3.695573064303025 | 4.2726195945023155 |
| Guaiacyl (9) | 3.8269997871299397 | 28.296818639280744 | 30.549124484229957 |
| Guaiacyl (10) | 1.8937158851899627 | 18.86305026998899 | 15.672120482226886 |
| Syringyl (11) | 1.2371300985556608 | 4.876440240451361 | 6.529643792756664 |
| Syringyl (12) | 1.5553585271933559 | 6.9690582795110965 | 7.420910569509455 |
| p-Hydroxyphenyl (13) | 0.8460468917794031 | 2.5875194744377414 | 2.286335663351667 |
| Cinnamyl alcohol (14) | 1.067498077815127 | 0.664375301067213 | 1.0213041480948064 |
| Ferulate (15) | 1.3234365964936807 | 3.7046998638515376 | 2.3814658008765504 |
| Ferulate (16) | 2.451631633126394 | 7.897552128764647 | 5.708975329947978 |
| Methoxyl (17) | 21.72071606946554 | 102.27182744691453 | 104.31068456186965 |
| Methoxyl (18) | 37.96791063270065 | 154.1923161137682 | 159.60670945954334 |
| 5-5/4-O-β (19) | 1.7882514789703443 | 0.7915385959783535 | 1.3929102053147595 |
| 5-5/4-O-β (20) | 0.6536604118876809 | 0.035389526234124065 | 0.031594717521489554 |
| β-O-4 (21) | 1.700207468879668 | 2.182934060510515 | 2.6038170087199797 |
| β-O-4 (22) | 1.3739420040286454 | 1.9735080578506692 | 2.2441846798190617 |
| β-O-4 (23) | 1.4834521328309176 | 1.6610376714233808 | 1.8750307289274053 |
| β-O-4-H/G (24) | 85.15681851397635 | 12.297748914777218 | 13.203906815820948 |
| β-O-4-H/G (25) | 1.7032273642873936 | 6.300420596567765 | 22.15397323996706 |
| β-O-4-H/G (26) | 1.3163276896290979 | 1.809823756585013 | 1.9801489059674129 |
| β-O-4-H/G (27) | 1.4589617501096122 | 1.8274863664141352 | 2.0659010300915868 |
| β-O-4-S (28) | 3.326646327514885 | 0.3696365652713718 | 0.47920034432952996 |
| β-5 (29) | 0.6268117783863814 | 1.425653116127024 | 1.701969134499584 |Raw biomass
1-butanol
1-pentanol
Additional_file_2 as PPTX
