# Supplementary material for: Organosolv pretreatment of sorghum bagasse using a low concentration of hydrophobic solvents such as 1-butanol or 1-pentanol
Source: Biotechnol Biofuels. 2016 Feb 2;9:27. doi: 10.1186/s13068-016-0427-z (PMC4736640; doi:10.1186/s13068-016-0427-z)
Supplement: Supplementary file 3 — 10.1186/s13068-016-0427-z Peak intensities obtained by 2D NMR analysis of solid fractions. The solid fractions were obtained by organosolv pretreatment of sorghum bagasse using 1-butanol and 1-pentanol as the solvent and no solvent (control). Red, orange, and blue bars indicate solid fraction pretreated with no solvent, 1-butanol, and 1-pentanol, respectively. [file 13068_2016_427_MOESM3_ESM.pptx]

## Slide 1
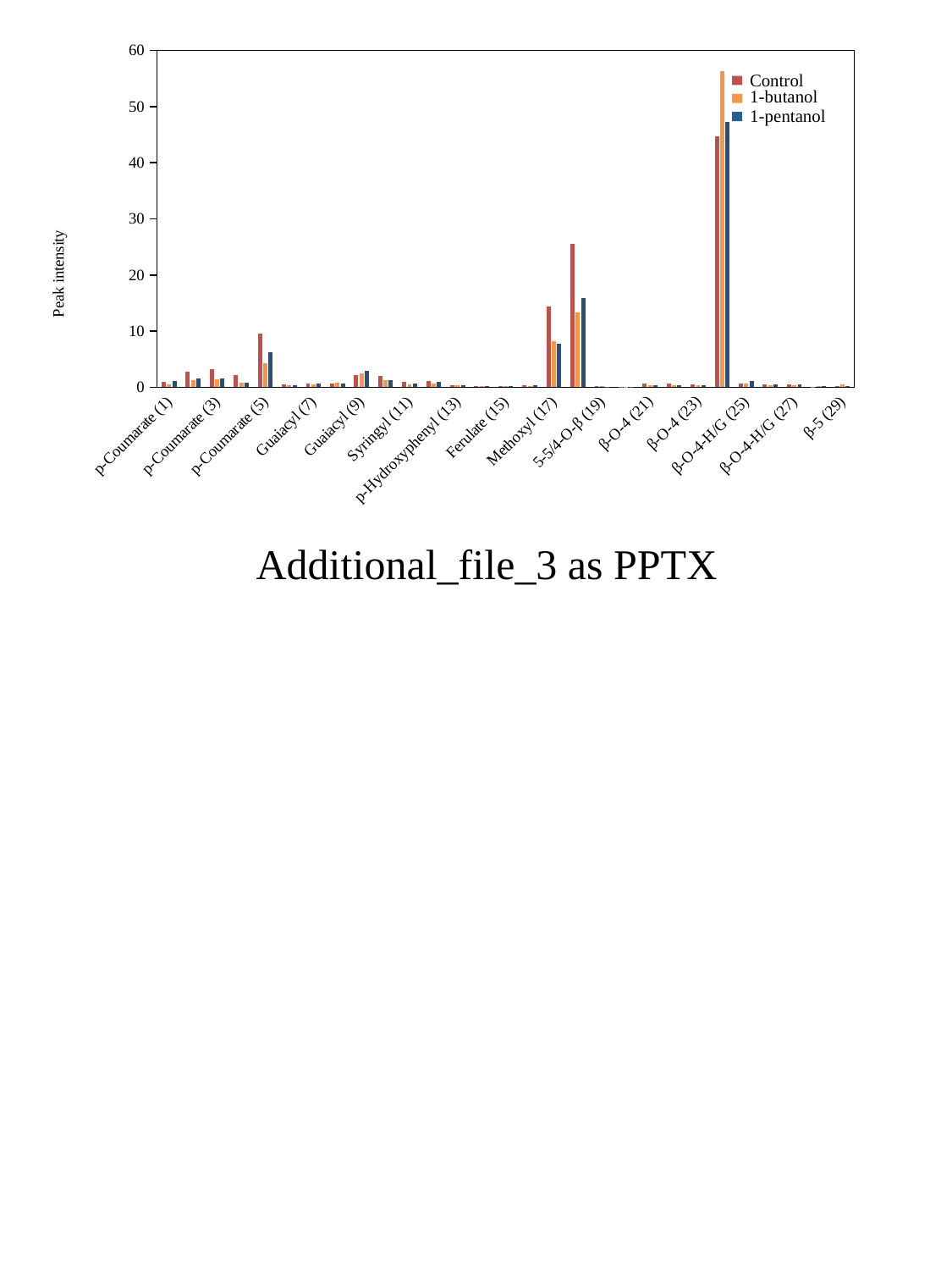

### Chart
| Category | No302_sorghum_H2SO4 | No305_sorghum_H2SO4_BuOH | No314_1_sorghum_H2SO4_PeOH_sol |
|---|---|---|---|
| p-Coumarate (1) | 0.9726867263557921 | 0.5750654643329633 | 1.049791603563103 |
| p-Coumarate (2) | 2.796936059086299 | 1.3053867793981235 | 1.6044333268593336 |
| p-Coumarate (3) | 3.2612697409650226 | 1.4060887151959367 | 1.6278938644236858 |
| p-Coumarate (4) | 2.1777085729946566 | 0.7587822569608126 | 0.7472799786089006 |
| p-Coumarate (5) | 9.536434398113316 | 4.22638372287526 | 6.192261113445458 |
| p-Coumarate (6) | 0.5081086553886921 | 0.29668526938458967 | 0.33052611021473416 |
| Guaiacyl (7) | 0.7365396599849526 | 0.5838445279116802 | 0.7346427670269331 |
| Guaiacyl (8) | 0.6715833096217729 | 0.8692662824470431 | 0.6498937170101742 |
| Guaiacyl (9) | 2.1571247274625254 | 2.4006928118489683 | 2.9322204509885044 |
| Guaiacyl (10) | 2.0242563152758892 | 1.2501391402198871 | 1.3353434116113427 |
| Syringyl (11) | 0.935085719472748 | 0.5393521996320008 | 0.6659217083611976 |
| Syringyl (12) | 1.0688135084322963 | 0.6624549505025775 | 0.9690095372237947 |
| p-Hydroxyphenyl (13) | 0.3341149711009903 | 0.3639169112391843 | 0.35862156530553063 |
| Cinnamyl alcohol (14) | 0.23484901118429208 | 0.2582044068672922 | 0.19515665913638278 |
| Ferulate (15) | 0.17142183825981003 | 0.16950928361794984 | 0.14275599782000165 |
| Ferulate (16) | 0.2935011704491697 | 0.19020209546691808 | 0.31912819434050477 |
| Methoxyl (17) | 14.374576552263479 | 8.16943294657928 | 7.687108903614698 |
| Methoxyl (18) | 25.614755514901624 | 13.379892641535257 | 15.92736495120021 |
| 5-5/4-O-β (19) | 0.14567335303574888 | 0.16607989537871992 | 0.13036142672606965 |
| 5-5/4-O-β (20) | 0.07834450831533873 | 0.09928194522589377 | 0.05632191452491154 |
| β-O-4 (21) | 0.6499585411367933 | 0.3617989385806177 | 0.3220183599762632 |
| β-O-4 (22) | 0.6245134475095879 | 0.3617989385806177 | 0.3572625633703248 |
| β-O-4 (23) | 0.4969088271593085 | 0.3240324812578884 | 0.3066480803694161 |
| β-O-4-H/G (24) | 44.71673733584472 | 56.38773133011968 | 47.3276738739679 |
| β-O-4-H/G (25) | 0.5932541629062751 | 0.6923626465534283 | 1.1780679899091686 |
| β-O-4-H/G (26) | 0.489205508530052 | 0.3452642143519715 | 0.46090851915595543 |
| β-O-4-H/G (27) | 0.5287429498112094 | 0.3499165158680677 | 0.46090851915595543 |
| β-O-4-S (28) | 0.08149346171906777 | 0.16379951643071122 | 0.14962283123251147 |
| β-5 (29) | 0.17265198819251576 | 0.47496358023752683 | 0.21652193421591345 |Control
1-butanol
1-pentanol
Additional_file_3 as PPTX
